# Leaf powder supplementation of *Senna alexandrina* ameliorates oxidative stress, inflammation, and hepatic steatosis in high-fat diet-fed obese rats

**DOI:** 10.1371/journal.pone.0250261

**Published:** 2021-04-20

**Authors:** Shariful Islam Nayan, Faizul Islam Chowdhury, Noushin Akter, Md Mizanur Rahman, Saima Selim, Nadia Saffoon, Ferdous Khan, Nusrat Subhan, Maqsud Hossain, K. Shahin Ahmed, Hemayet Hossain, Md Areeful Haque, Md Ashraful Alam

**Affiliations:** 1 Department of Pharmaceutical Sciences, North South University, Dhaka, Bangladesh; 2 Department of Pharmacy, International Islamic University Chittagong, Chittagong, Bangladesh; 3 NSU Genome Research Institute (NGRI), North South University, Dhaka, Bangladesh; 4 BCSIR Laboratories, Bangladesh Council of Scientific and Industrial Research, Dhaka, Bangladesh; State University of Rio de Janeiro, BRAZIL

## Abstract

Obesity is an enduring medical issue that has raised concerns around the world. Natural plant extracts have shown therapeutic potential in preventing oxidative stress and inflammation related to obesity complications. In this study, *Senna alexandrina* Mill. leaves were utilized to treat high-fat diet-related metabolic disorders and non-alcoholic fatty liver diseases. Plasma biochemical assays were conducted to determine the lipid profiles and oxidative stress parameters, and the gene expression of antioxidant enzymes and inflammatory mediators was measured. Histological stained livers of high-fat diet-fed rats were observed. *S*. *alexandrina* leaf powder supplementation prevented the increase in cholesterol and triglyceride levels in high-fat diet-fed rats. Moreover, *S*. *alexandrina* leaves also reduced lipid peroxidation and nitric oxide production in these rats. Prevention of oxidative stress by *S*. *alexandrina* leaf supplementation in high-fat diet-fed rats is regulated by enhancing the antioxidant enzyme activity, followed by the restoration of corresponding gene expressions, such as *NRF-2*, *HO-1*, *SOD*, and *CAT*. Histological staining provides further evidence that *S*. *alexandrina* leaf supplementation prevents inflammatory cell infiltration, lipid droplet deposition, and fibrosis in the liver of high-fat diet-fed rats. Furthermore, this investigation revealed that *S*. *alexandrina* leaf supplementation controlled non-alcoholic fatty liver disease by modulating the expression of fat metabolizing enzymes in high-fat diet-fed rats. Therefore, *S*. *alexandrina* leaf supplementation inhibits fatty liver inflammation and fibrosis, suggesting its usefulness in treating non-alcoholic steatohepatitis. Thus, this natural leaf extract has potential in treatment of obesity related liver dysfunction.

## Introduction

Obesity leads to several life-threatening diseases, such as glucose intolerance, hypertension, cardiovascular diseases, liver dysfunction, and many types of cancer. It is now considered a global epidemic that cuts across gender and age. In 2016, over 650 million adults were classified as obese, which is approximately three times more than in 1975. The percentage of adults in the USA classified as obese is 35.7%, while the percentage of youth classified as such is 16.9% [1]. Another study estimated that the number of obese adults in the USA and UK would be 65 million and 11 million, respectively, by 2030 [2].

Epidemiological studies indicate that about 80% of obese people develop non-alcoholic fatty liver disease (NAFLD). Among them, 25% are at high risk of developing non-alcoholic steatohepatitis, and 42% are likely to develop advanced fibrosis, cancer or liver failure [3].

The pathophysiology of obesity is complex, but it is believed that regular intake of high-fat (HF) food, along with low levels of physical activity, is the main reason for its development [4]. Mass storage of triglycerides in fat cells due to HF diet results in hypertrophy of the white adipose tissue which induces hypoxia and secretes pro-inflammatory adipokines like tumor necrosis factor-α (TNF-α), interleukin-6 (IL-6), and IL-1β [5]. HF diet may also lead to the deposition of fat in the liver, steatosis and the development of non-alcoholic fatty liver diseases (NAFLD) [4, 6]. Moreover, HF diet also modulates the transcriptional factors for fat metabolism in liver such as sterol regulatory element-binding protein 1c (SREBP-1c), fatty acid synthase (FAS), CEBPα and peroxisome proliferator-activated receptor gamma (PPAR-γ) [4]. HF diet may also induce PPARγ activation in liver which has been proposed as a regulator for fatty acid deposition as lipid droplets in hepatocytes [7].

Although the pathological mechanism of NAFLD is complex, many researchers have used the “two-hit” hypothesis to describe it (hepatic steatosis is set as “first-hit” and oxidative stress and inflammation are known as “second-hit”) [6, 8]. Recently, researchers have identified important factors, such as cytokine release (pro-inflammatory TNF-α and IL-6), aggravated inflammatory response mediated by Kupffer cells (liver macrophages), endoplasmic reticulum stress, and direct hepatic cell lipotoxicity in the pathogenesis of NAFLD [6, 9]. Treatment approaches for steatosis or NAFLD, which is strongly linked with obesity, can be non-pharmacological and pharmacological. Non-pharmacological approaches aimed at reducing body weight via lifestyle modification, exercise, low-calorie intake, and a diet rich in monounsaturated and polyunsaturated fats [6]. Pharmacological approaches include the use of lipid-lowering drugs (e.g., atorvastatin) and drugs that can help in weight loss (e.g., orlistat). Furthermore, oxidative stress, which leads to peroxidation and damage to the hepatic tissue; thus, antioxidants like vitamin E and cytoprotective agents are also suggested as part of the treatment [10].

*Senna alexandrina* Mill. or Alexandrian senna, a versatile medicinal plant distinguished for its laxative effects, is distributed throughout the subtropical and tropical regions of the world (Pakistan, India, Saudi Arabia, Africa, Mexico) [11]. It is also called *Cassia angustifolia* Vahl., *Cassia acutifolia* Delie., *Cassia senna* L., *Cassia lanceolata* Forssk., *Senna angustifolia*, and *Senna acutifolia* [12]. Studies on the antidiabetic and hypoglycemic effects of senna suggest that it might be a novel antihyperglycemic agent for the treatment of diabetes mellitus, especially type 2 diabetes. Extracts from different parts of senna showed considerable hypoglycemic effects in different animal models. Anthraquinone glycosides such as sennoside-A, sennoside-B, and saponin exhibit antihyperglycemic activity [13]. Flavonols such as rutin are listed as showing anti-diabetic activity [14]. These glycosides, saponin, and rutin, are found in high quantities in senna extract [15]. *S*. *alexandrina* Mill. is also used in some areas of Iran to lower blood lipid levels [16]. The aqueous extract of senna improves metabolic abnormalities and oxidative stress linked with diabetes, and reduces chronic hyperglycemia-related complications in rats [17]. *In vitro* studies on the anti-diabetic effects of senna revealed that different solvent extracts of *S*. *alexandrina* Mill. can inhibit these enzymes [18]. However, very few studies have been performed to understand the effect of *S*. *alexandrina* supplementation on HF diet-induced hepatic steatosis, hyperlipidemia and oxidative stress.

The treatment of obesity and its related complications is not easy. It is a time-consuming process, and sometimes patients express their unwillingness to participate in treatment approaches such as exercise. Very few drugs have been approved by the FDA to treat obesity or its related complications. The FDA recommends precautions against taking these drugs due to their undesirable side effects. Hence, alternative medicines, antioxidants, and polyphenolic-rich functional food might be good alternatives to treat obesity and its related complications [6, 19]. Considering the above discussion, this investigation was designed to assess the effect of *S*. *alexandrina* leaf powder supplementation on hepatic steatosis, hyperlipidemia and oxidative stress in HF diet fed rats.

## Materials and methods

### Chemicals

Alkaline phosphatase (ALP), alanine aminotransferase (ALT), cholesterol (total) liquid, triglyceride liquid, high-density lipoproteins, and low-density lipoprotein assay kits were collected from DCI diagnostics (Budapest, Hungary). Thiobarbituric acid was obtained from Sigma Chemical Company (USA). Reduced glutathione (GSH) was purchased from J.I. Baker (USA). All other chemicals and reagents used were of analytical grade.

### *S*. *alexandrina* leaf powder preparation

Leaves of *S*. *alexandrina* were collected from Savar, Bangladesh. After collection, all leaves were ground to fine powder and used as food supplements. The specimen was identified by an expert in the National Herbarium, Mirpur, Dhaka, and a voucher number was deposited in the Herbarium (Accession number: 47411).

### Animals

Ten to twelve weeks-old Wistar male rats (total n = 40) with bodyweight 185–215 g were collected from the Animal production unit of the Animal House at the Department of Pharmaceutical Sciences, North South University. All rats were kept in individual cages with free access to water and selected food. The environmental conditions in this facility were maintained at a temperature of 23 ± 2 °C and humidity of 52–55%, along with 12:12 h light/dark cycles.

The diet was classified into two food groups: HF diet and control diet. The HF diet was composed of condensed milk, sugar, beef tallow, and normal food. The caloric content of the HF diet contained approximately 49% fat, 14% proteins, and 37% carbohydrates. Control diet was made from corn starch, rice polishing, wheat, and wheat bran, where caloric content was approximately 60% carbohydrate, 15% fat, and 25% proteins [20].

### Study design

The experimental design was approved by the Ethical Committee of North South University for animal care and experimentation (AEC 009–2018). To assess the effect of *S*. *alexandrina* on high-fat diet-fed rats, the total number of rats was divided into five distinct groups (8 rats in each group):

Control (control food and water were given for 56 days); HF (high-fat diet was given for 56 days); control + SA (rats in this group received similar treatment as group 1 along with 1% of food, w/w of *S*. *alexandrina* leaf powder every day for 56 days); HF + SA (rats in this group received both HF diet and 1% of food, w/w of *S*. *alexandrina* leaf powder as a supplement every day during the study); and HF + ATO (rats received a HF diet along with 10 mg/kg of atorvastatin by oral gavage for 56 days.

An oral glucose tolerance test (OGTT) was performed on the first day and the day before sacrificing the rats. All five groups were tested to check the glycemic activity before and after consumption of HF diet. The body weight, food intake, and water intake of these 40 rats were recorded over 56 days.

### Sacrificing animals and collecting samples

On the 57^th^ day of the study, all rats were weighed and appropriately anesthetized using ketamine at a high dose (90 mg/kg) to euthanize the animals [21]. After sacrificing the animals, the blood sample was collected from the abdominal aorta and promptly stored in a tube with citrate buffer. Subsequently, the plasma was separated by centrifugation (8000 rpm for 15 min) and stored at -20 °C until future analysis. All the internal organs including liver, spleen, peritoneal fat, epididymal fat, and mesenteric fat, were separated on ice, weighed immediately, preserved in neutral buffered formalin for histological analysis, and stored in a refrigerator (at -20 °C) for further investigation.

### Oral glucose tolerance test

Prior to performing the OGTT, all the experimental animals were starved for 12 hours. During this time, only normal water supply was provided. Blood from the tail tip was taken to minimize stress and estimate basal blood glucose levels using a glucometer (Bionim Corporation, Bedford, MA, USA). Thereafter, a 40% aqueous solution of glucose was administered orally at a dose of 2 g/kg body weight. After glucose administration, blood glucose concentration was analyzed using a glucometer at different time intervals (30, 60, 90, and 120 min).

### Assessment of liver function markers

The activities of enzymes in plasma that mark liver function were estimated using DCI diagnostics kits (Budapest, Hungary) according to the manufacturer’s standard protocol. These enzymes include ALT, aspartate aminotransferase (AST), and ALP (Mamun, 2020).

### Assessment of lipid profiles

The plasma concentrations of cholesterol and triglycerides were measured using assessment kits supplied by DCI diagnostics. The tests were performed according to the standard protocol provided by the manufacturer.

### Assessment of oxidative stress markers

#### Preparation of tissue sample

Liver tissue (1 g) was homogenized in 10 mL of phosphate buffer (pH 7.4) and centrifuged at 8000 rpm for 15 min at 4 °C. The supernatant was separated in a fresh Eppendorf tube and used to assess proteins and conduct enzymatic studies, as described below.

#### Lipid peroxidation assessment

Plasma levels of thiobarbituric acid reactive substances indicate lipid peroxidation and oxidative stress. Hepatic lipid peroxidation was measured calorimetrically by estimating thiobarbituric acid reactive substances, as previously described [22].

#### Nitric oxide assay

Nitric oxide (NO) assay was performed following a previously described method [23]. NO concentration was estimated using a standard curve and displayed as mmol/g.

#### Assessment of advanced protein oxidation product

Advanced protein oxidation product (APOP) concentration was estimated using methods previously described by Witko-Sarsat [24] and [25]. APOP concentrations were displayed as μmol·L^−1^ chloramine-T equivalents.

### Superoxide dismutase and reduced glutathione antioxidant activity assays

Superoxide dismutase (SOD) activity was measured in hepatic tissue and plasma following a previously established method [26]. To determine SOD activity, we recorded changes in absorbance four times at 480 nm. The time interval of two consecutive readings was 15 s, and 50% neutralization of the antioxidant activity of adrenaline present in the assay was considered a unit of enzyme activity. We estimated the reduced GSH levels following the method described by Jollow et al. [27]. The absorbance was measured at 412 nm when a yellowish color was seen in the mixture. Concentration was expressed as ng/mg protein.

### Estimating the fat metabolizing, inflammation, and oxidative stress regulatory gene expressions

Liver tissue was chosen for the determination of total mRNA, which was isolated and purified using the GeneJet RNA purification kit from Thermo-Fisher Scientific. To measure the concentration of the total mRNA, NanoDrop 2000 spectrophotometer was used. From each sample, 1 μg mRNA was taken and cDNA synthesis was performed using the Revert AID First strand cDNA Synthesis kit. To measure the transcript levels of metabolization, SYBR Premix Ex Taq was used for quantitative real-time polymerase chain reaction. This quantification was analyzed following the manufacturer’s protocol using Thermal Cycler Real-Time Single. Forward and reverse primers, which were used for quantitative real-time PCR, were designed using primer3 online software (Table 1). The first step of polymerase chain reaction was 95 °C for 1 minute, followed by 40 cycles of amplification for 5 seconds at 95 °C for denaturation. Annealing was performed at 60 °C for 30 s, extension was done for 1 min at 72 °C, and the final extension was performed for 5 min at the same temperature.

**Table 1:** The forward and reverse sequence of the primer used in this study.

Name of gene (Ref. sequence no.)	Type	Sequence	T_M_ Value
Nrf-2 (XM_213329)	Forward	5′-CCCAGCACATCCAGACAGAC-3′	60.2°C
	Reverse	5′-TATCCAGGGCAAGCGACTC-3′	58.9°C
HO-1 (NM 012580.2)	Forward	5′-TGCTCGCATGAACACTCTG-3′	59.2°C
	Reverse	5′-TCCTCTGTCAGCAGTGCCT-3′	58.7°C
HO-2 (J05405.1)	Forward	5′-CACCACTGCACTTTACTTCA-3′	58.3°C
	Reverse	5′-AGTGCTGGGGAGTTTTAGTG-3′	57.9°C
MnSOD NM_017051.2	Forward	5′-GCTCTAATCACGACCCACT-3′	59.2°C
	Reverse	5′-CATTCTCCCAGTTGATTACATTC-3′	58.7°C
Catalase AH004967.2	Forward	5′-ATTGCCGTCCGATTCTCC-3′	59.3°C
	Reverse	5′-CCAGTTACCATCTTCAGTGTAG-3′	60.1°C
GPx S50336.1	Forward	5′-AGAATGAAGAGATTCTGAAT-3′	58.7°C
	Reverse	5′-GGACGGCTTCATCTTCAGTGA-3′	58.6°C
IL-1 M98820	Forward	5′-ATGCCTCGTGCTGTCTGACC-3′	59.3°C
	Reverse	5′-CCATCTTTAGGAAGACACGGGTT-3′	58.7°C
IL-6 M26744	Forward	5′-AGCGATGATGCACTGTCAGA-3′	58.9°C
	Reverse	5′-GGTTTGCCGAGTAGACCTCA-3′	59.2°C
TNF-α L00981.1	Forward	5′-ATGTGGAACTGGCAGAGGAG-3′	59.1°C
	Reverse	5′-CCACGAGCAGGAATGAGAAGAG-3′	57.9°C
TGF-β NM_021578.2	Forward	5′-AAGAAGTCACCCGCGTGCTA-3′	59.2°C
	Reverse	5′-TGTGTGATGTCTTTGGTTTTGTC-3′	60.3°C
iNOS NM_012611.3	Forward	5′-TGGTCCAACCTGCAGGTCTTC-3′	59.4°C
	Reverse	5′-CAGTAATGGCCGACCTGATGTTG-3′	58.3°C
NF-кB NM_001276711.1	Forward	5′-TGTGAAGAAGCGAGACCTGGAG-3′	60.5°C
	Reverse	5′-GGCACGGTTATCAAAAATCGGATG-3′	59.3°C
β-Actin V01217.1	Forward	5′-GCGAGAAGATGACCCAGATC-3′	58.4°C
	Reverse	5′-GGATAGCACAGCCTGGATAG-3′	59.4°C
SREBP-1c NM_001276707.1	Forward	5′-GGCATGAAACCTGAAGTGGT-3’	58.3°C
	Reverse	5′-TGCAGGTCAGACACAGGAAG-3’	58.1°C
FAS (M76767.1)	Forward	5’-TCGAGACACATCGTTTGAGC-3’	60.7°C
	Reverse	5’-CTCAAAAAGTGCATCCAGCA-3’	59.6°C
PPARγ2 AB019561.1	Forward	5’-CCCTGGCAAAGCATTTGTAT-3’	59.3°C
	Reverse	5’-GAAACTGGCACCCTTGAAAA-3’	58.9°C
C/EBPα NM_001287579.1	Forward	5’-GCCAAGAAGTCGGTGGATAA-3’	57.9°C
	Reverse	5’- CCTTGACCAAGGAGCTCTCA-3’	59.2°C
Leptin (NM_013076.3)	Forward	5′-GAGACCTCCTCCATCTGCTG-3’	58.7°C
	Reverse	5′-CATTCAGGGCTAAGGTCCAA-3’	60.1°C
Adiponectin (NM144744.3)	Forward	5’-AATCCTGCCCAGTCATGAAG-3’	58.8°C
	Reverse	5’-TCTCTCCAGGAGTGCCATCT-3’	59.6°C

### Histopathology

To investigate any changes in liver tissue, microscopic evaluation was performed. Liver tissues were processed in neutral buffered formalin and embedded in paraffin to arrange the mold. Then, the molded tissues were sharply sliced to 5 μm. Subsequently, these thin slices were stained with three different staining processes for investigating three different characteristics: a) Hematoxylin-eosin to observe liver tissue layout, fat deposition, and inflammatory cell infiltration; b) Sirius red staining to detect fibrosis in liver; c) Periodic acid schiff staining on intestine to see the Goblet cell population. Sections were then studied and photographed using a microscope (Zeiss Axioscope) at 40 × magnification. ImageJ 1.50i software from National Institute of Health (NIH), USA was used for steatosis and fibrosis analysis. The image was converted to RGB stacks and montage, and then threshold was adjusted manually. The result was expressed as % of area. Histology slides were prepared from at least three rats from each group. Five random snaps were taken from each slide and analyzed for the steatosis and fibrosis score [28].

### Statistical analysis

The mean ± SEM was calculated for all values. In case of gene expression, mean ± SD was used. Statistical analysis was performed by one-way ANOVA, and comparisons among the groups were performed using Newman-Keul’s multiple comparisons test using the Graph Pad Prism software system, version 6. Values are considered significantly different only when p < 0.05.

## Results

### Effects of HF diet and *S*. *alexandrina* leaf powder supplementation on body weight and food and water intake

Table 2 shows initial and final body weight as well as body weight gain of all groups of rats. We observed that the final body weight of rats of the HF group was significantly higher (p ≤ 0.001) than that of rats from the control group. On the other hand, supplementation with *S*. *alexandrina* leaf powder resulted in significantly lower final body weight (p ≤ 0.001) in the HF + SA group compared to that in HF group. This reduction in body weight was comparable to that seen in HF + ATO rats (Table 2). However, *S*. *alexandrina* leaf powder supplementation also resulted in reduced body weight gain in control + SA rats (Table 2).

**Table 2:** Effects of HF diet and *Senna alexandrina* leaf powder supplementation on initial body weight, final body weight, food intake and water intake.

Parameters	Control	HF	Control+SA	HF+SA	HF+ATO
**Initial body weight (g)**	186±2.01	201.375±1.99	188.75±2.95	211±3.52	189.25±3.44
**Final body weight (g)**	208.75±2.97**	294±6.72#	160.5±2.79**	214.25±5.98**	204.25±5.87**
**Food intake (g)/d**	28.26±0.63	18.20±1.06	22.41±2.16	16.76±1.41	14.09±1.62
**Water intake mL/d**	20.83±3.53	26.06±2.42	16.43±1.46	23.77±2.02	18.14±1.84

Data are presented as mean ± SEM, n = 8. For statistical analysis, one-way ANOVA with Tukey’s multiple comparison test was performed.

** Versus

^#^ represents p≤0.01.

Body weight gain depends on food intake and water intake. Hence, we measured food intake and water intake of each group (Table 2). Rats in the control group consumed more food than the rats from the HF group. However, when we compared HF group and HF + SA group for the consumption of food, no significant difference was observed in food consumption. Contrarily, atorvastatin affected the food intake in HF + ATO rats, which may explain the reduction in body weight in this group (Table 2), whereas *S*. *alexandrina* leaf powder supplementation did not affect food consumption in control + SA rats (Table 2). Water intake was not significantly different among these groups.

### Effect of *S*. *alexandrina* leaf powder supplementation on liver weight and fat deposition in liver

**Fig 1:**
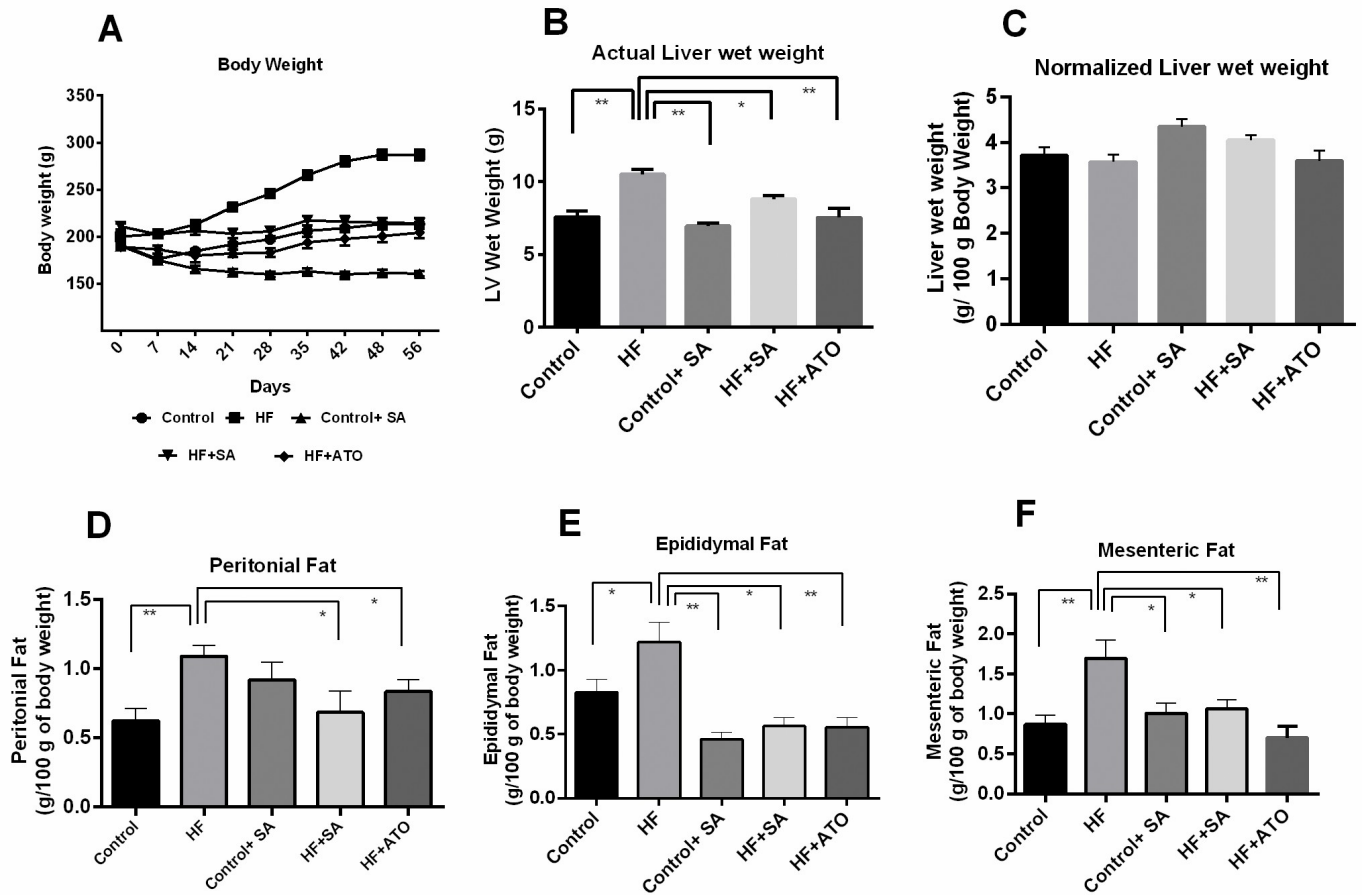
Effect of *Senna alexandrina* leaf powder supplementation on body weight and fat deposition in high-fat diet-induced obese rats. A. body weight gain; B. Actual liver wet weight; C. Normalized liver wet weight; D. Peritoneal Fat; E. Epididymal Fat and F. Mesenteric Fat deposition in high fat diet induced obese rats. Data are presented as mean ± SEM, n = 8. For statistical analysis, one-way ANOVA with Tukey’s multiple comparison test was performed. * represents p ≤ 0.05; ** represents p ≤ 0.01.

At the end of the treatments, vital organs were obtained from euthanized animals and wet weights were determined. The organ weight data are presented in Fig 1. These results showed that the actual liver wet weight of rats in the HF group was significantly higher (p ≤ 0.01) than that of the control group (Fig 1B). However, actual liver wet weight of the HF + SA group was significantly lower (p ≤ 0.05) than that of the HF group, indicating that *S*. *alexandrina* prevented liver enlargement (Fig 1B). This result is also comparable to the atorvastatin treatment in HF + ATO rats, which also resulted in a lower liver wet weight (Fig 1B). *S*. *alexandrina* treatment in control + SA rats also resulted in a lower liver wet weight compared to the HF rats (Fig 1B). However, the normalized wet weight of liver in control + SA rats was significantly higher than that in HF rats (Fig 1C). This discrepancy occurred because of the substantial lowering of body weight in control rats treated with *S*. *alexandrina*. However, the normalized values of liver wet weights of the other groups were not significantly different.

Fig 1D, 1E and 1F display the accumulation of peritoneal, epididymal, and mesenteric fats across all the groups. Peritoneal fat deposition in the HF group was significantly higher (p ≤ 0.001) than that in the control group (Fig 1D). *S*. *alexandrina* supplementation and atorvastatin treatment significantly decreased peritoneal fat (p ≤ 0.05) deposition in HF + SA and HF + ATO rats (Fig 1D). However, rats in the control + SA group showed no alteration in peritoneal fat deposition compared to those in the HF group (Fig 1D).

Rats in the control group showed significantly lower epididymal fat deposition than those in the HF group (p ≤ 0.05) (Fig 1E). However, *S*. *alexandrina* and atorvastatin significantly reduced epididymal fat deposition (p < 0.05) in HF diet-fed rats (Fig 1E). *S*. *alexandrina* also reduced epididymal fat deposition in control rats (Fig 1E).

Mesenteric fat deposition is directly linked to the supply of free fatty acids to the liver. We observed that HF diet significantly increased mesenteric fat deposition (p ≤ 0.001) relative to the control group (Fig 1F). Additionally, *S*. *alexandrina* and atorvastatin significantly prevented mesenteric fat deposition in HF diet-fed rats (Fig 1F). Mesenteric fat deposition was also significantly reduced in control + SA group compared to control group (Fig 1F). Hence, *S*. *alexandrina* reduces fat deposition in HF diet-fed rats.

### Effect of *S*. *alexandrina* leaf powder supplementation on oral glucose tolerance test

**Fig 2:**
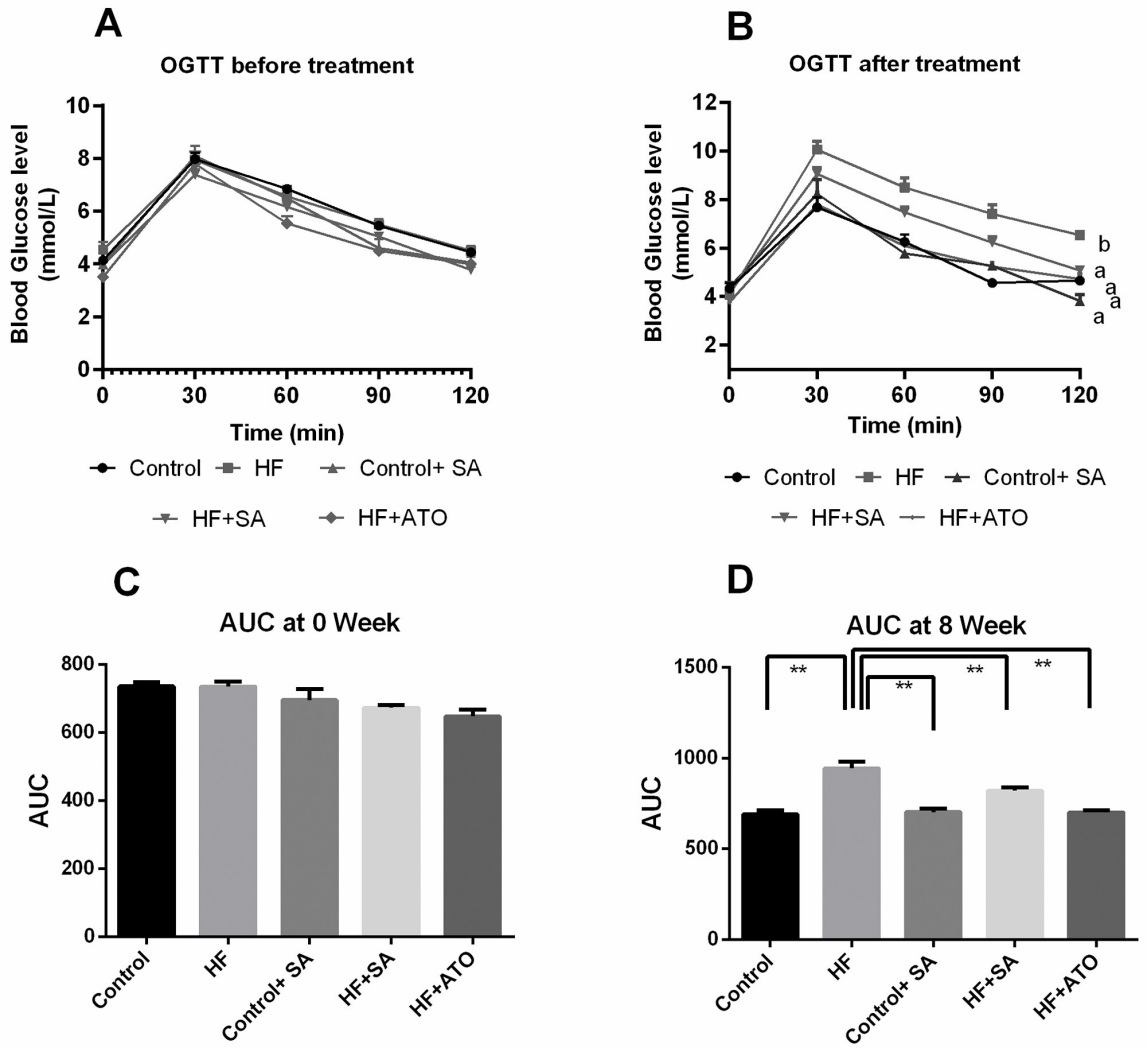
Effect of *Senna alexandrina* leaf powder supplementation on Oral Glucose Tolerance Test (OGTT) performed on the very first day and the day before sacrifice of the rats. Here, A. OGTT before treatment, B. OGTT after treatment, C. AUC at 0 week, and D. AUC at 8 week. Mean ± standard error of mean (SEM) was used to present all values, where n = 8. Statistical analysis was done by one-way ANOVA and comparisons among the groups were done following Tukey’s multiple comparisons test. Here, a vs b is significantly different at p<0.05.

Fasting blood glucose levels are usually high in obese subjects. Therefore, OGTT was performed to assess the glucose metabolizing ability of the experimental animals after consuming a high-fat diet for 56 days. In this study, the OGTT was performed twice, one on the very first day of the experiment and the other on the day before sacrificing the rats. The data of the oral glucose tolerance test of all groups are shown in Fig 2.

The result of the first OGTT showed that the initial plasma glucose concentration of all groups was approximately 4 mmol/L (Fig 2A). After oral glucose administration, the plasma glucose concentration of each group increased by nearly 8 mmol/L after 30 min and gradually returned to the normal level within 120 min. The area under the curve did not show any significant difference across the groups at this stage (Fig 2C).

However, the result of the second OGTT was different in the HF group, whereas the results of all other groups were almost the same (Fig 2B). Before rising at 30 min, the glucose concentrations were approximately the same for all 5 groups. The plasma glucose level generally started to decrease in the groups after 60 min and almost reached normal levels within 120 min. However, in the HF group, the plasma glucose level continued to remain high until 120 min. Plasma glucose levels in the HF + SA group reduced after 60 min of glucose load (Fig 2B). The area under the curve at 8 weeks was significantly (p ≤ 0.01) lower in the control group compared to that in the HF groups (Fig 2D). Again, *S*. *alexandrina* leaf powder supplementation significantly lowered the area under the curve (p ≤ 0.01) in the HF + SA group (Fig 2D), which was similar to the control group. Atorvastatin also resulted in a significant reduction of the area under the curve (p ≤ 0.01) in the HF + ATO group compared to the HF group, similar to that seen in HF + SA group. *S*. *alexandrina* treatment in control rats resulted in no abnormal changes in the OGTT test (Fig 2D). Thus, *S*. *alexandrina* supplementation mitigates the oral glucose tolerance that develops in rats given a high-fat diet.

### Effect of *S*. *alexandrina* leaf powder supplementation on hepatic marker enzymes

**Fig 3:**
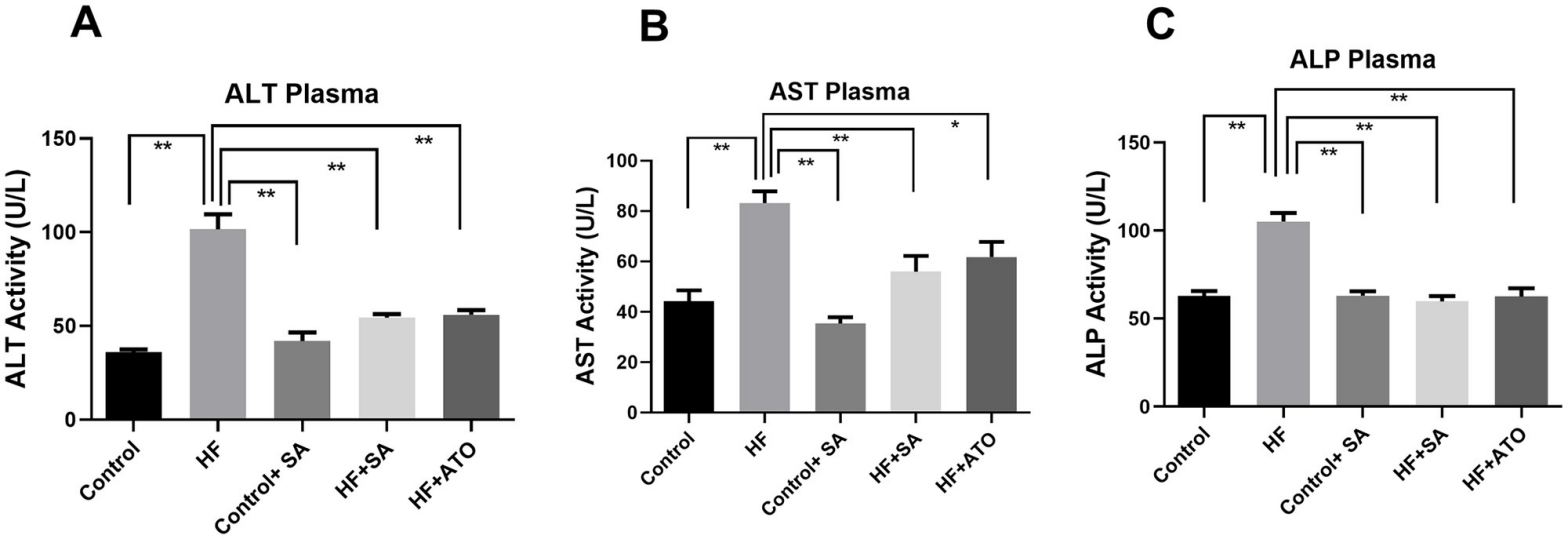
Effect of *Senna alexandrina* leaf powder supplementation on hepatic marker enzymes. A. ALT Plasma. B. AST Plasma. C. ALP in plasma. Mean± standard error of mean (SEM) was used to express all values, where n = 8. Statistical analysis was done by one-way ANOVA and comparisons among the groups were done following Tukey’s multiple comparisons test. * represents p≤0.05; ** represents p≤0.001.

The rate of fat accumulation in the liver is high in HF diet-fed rats, and can result in hepatic damage. To assess hepatic damage, the activities of liver marker enzymes such as ALT, AST, and ALP were measured. Changes in hepatic enzyme activity are shown in Fig 3.

ALT activity in HF group was significantly higher (p ≤ 0.01) than that in control group (Fig 3A). On the other hand, *S*. *alexandrina* leaf powder supplementation for 56 days lowered the ALT activity (p ≤ 0.01) in HF + SA group compared to HF group, such that that they were similar to the control group (Fig 3A). ALT activity in the HF + SA group was also comparable with that in HF + ATO group with a similar reduction in ALT activity (p ≤ 0.01) compared to the HF group (Fig 3A). AST and ALP enzyme activities in the plasma were also found to be approximately 2-fold higher in the rats of the HF group than in those of the control group (Fig 3B and 3C). However, AST and ALP activities were significantly lower in the HF + SA group (p ≤ 0.01) compared to those in the HF group. Atorvastatin treatment in the HF + ATO group also showed similar activity levels of these enzymes as seen in the HF + SA group (Fig 3B and 3C). Importantly, *S*. *alexandrina* supplementation did not affect the liver enzyme activities in control rats (Fig 3A, 3B and 3C). Thus, *S*. *alexandrina* supplementation reduces the activity of liver enzymes that increase due to HF diet.

### Effect of *S*. *alexandrina* leaf powder supplementation on oxidative stress markers

**Fig 4:**
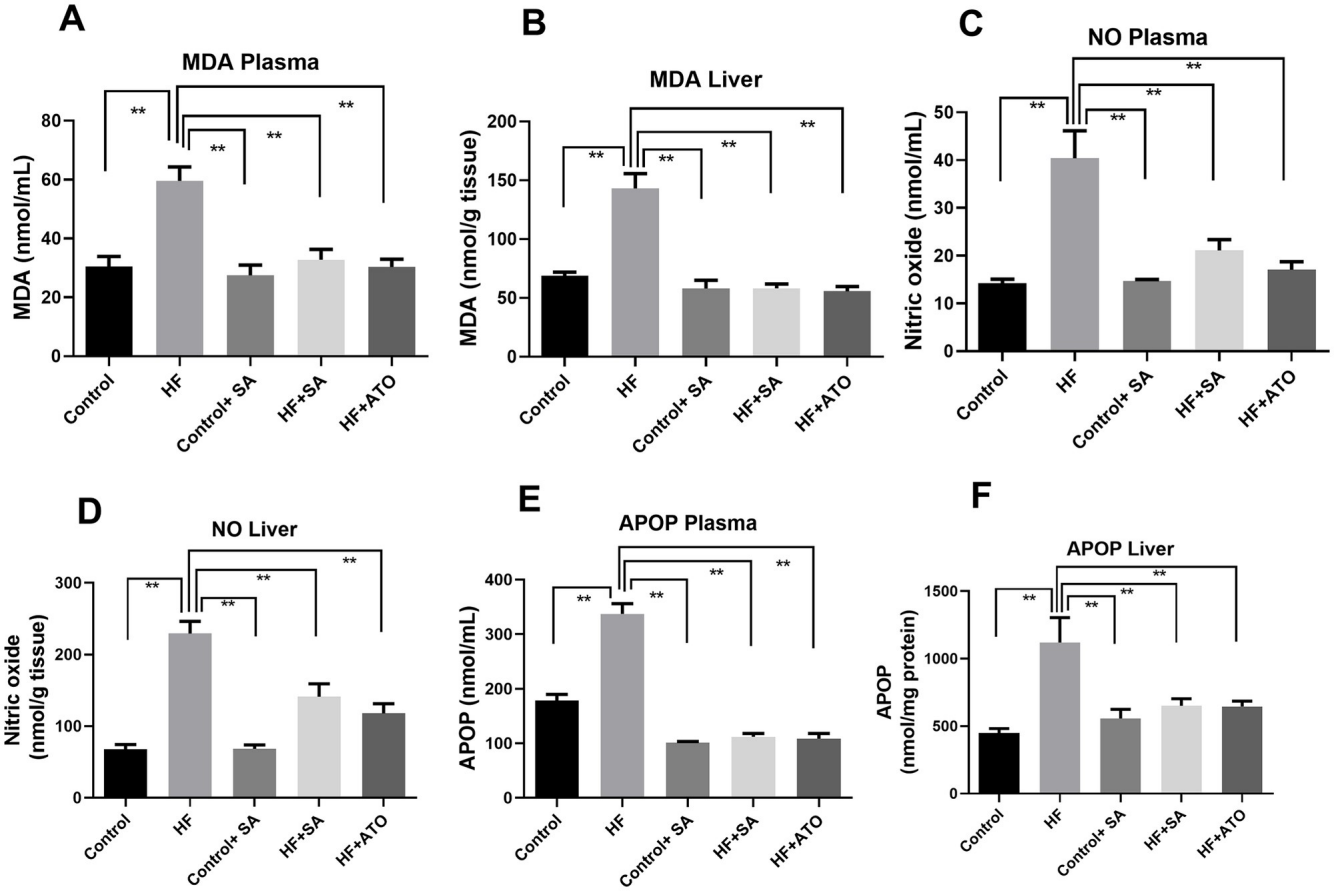
Effect of *Senna alexandrina* leaf powder supplementation on oxidative stress markers. A. MDA in plasma. B. MDA in liver. C. NO in plasma. D. NO in liver. E. APOP in plasma. F. APOP in liver. Mean ± standard error of mean (SEM) was used to express all values, where n = 8. Statistical analysis was done by one-way ANOVA and comparisons among the groups were done following Tukey’s multiple comparisons test. ** represents p≤0.001.

Oxidative stress was assessed by measuring the concentrations of malondialdehyde, APOP, and NO. The results of the different groups are shown in Fig 4.

Malondialdehyde concentrations in both the plasma and liver tissue were significantly (p ≤ 0.01) higher in the HF group than in the control group (Fig 4A and 4B). *S*. *alexandrina* supplementation reduced the increase in malondialdehyde concentration (p ≤ 0.01) in plasma and in the liver tissue of HF + SA rats. This result is also comparable to atorvastatin treatment in HF + ATO rats, which also showed an attenuation of lipid peroxidation in both the plasma and tissue (Fig 4A and 4B). However, *S*. *alexandrina* supplementation did not affect the malondialdehyde concentration in control rats (Fig 4A and 4B).

NO levels in both plasma and tissue were elevated more than 2-fold (p ≤ 0.01) in rats of HF group than in those of control group (Fig 4C and 4D). NO concentration was significantly reduced (p ≤ 0.01) in HF + SA rats. Similar reduction in NO concentration was seen in plasma and tissue of HF + ATO rats (Fig 4C and 4D). Moreover, *S*. *alexandrina* supplementation did not change the nitric oxide levels in either the plasma or liver tissue of control + SA rats (Fig 4C and 4D).

HF group showed increased APOP plasma concentration (Fig 4E) compared to control group. However, APOP plasma concentration was significantly reduced (p ≤ 0.01) in HF + SA rats compared to HF rats. Similarly, APOP plasma concentration level was significantly reduced (p ≤ 0.01) in HF + ATO group compared to the HF group (Fig 4E). However, *S*. *alexandrina* leaf powder supplementation treatment did not affect the plasma concentration of APOP in control + SA rats (Fig 4E).

HF rats also showed higher concentrations of APOP in liver tissue (p ≤ 0.01) compared to those of the control group (Fig 4F). However, the concentration of APOP was lower in HF + SA group (p ≤ 0.01) (Fig 4F) compared to HF group. The reduction of APOP concentration in liver tissue by *S*. *alexandrina* leaf powder supplementation was comparable with the effects of atorvastatin treatment (Fig 4F). APOP concentration in liver tissue was significantly reduced (p ≤ 0.01) in HF + ATO group compared to HF group. Moreover, control rats that were treated with *S*. *alexandrina* leaf powder supplementation showed normal levels of APOP concentration in liver tissue (Fig 4F). Hence, *S*. *alexandrina* supplementation ameliorates the increase in oxidative stress markers seen in rats fed with HF diet.

### Effect of *S*. *alexandrina* leaf powder supplementation on antioxidant enzymes

**Fig 5:**
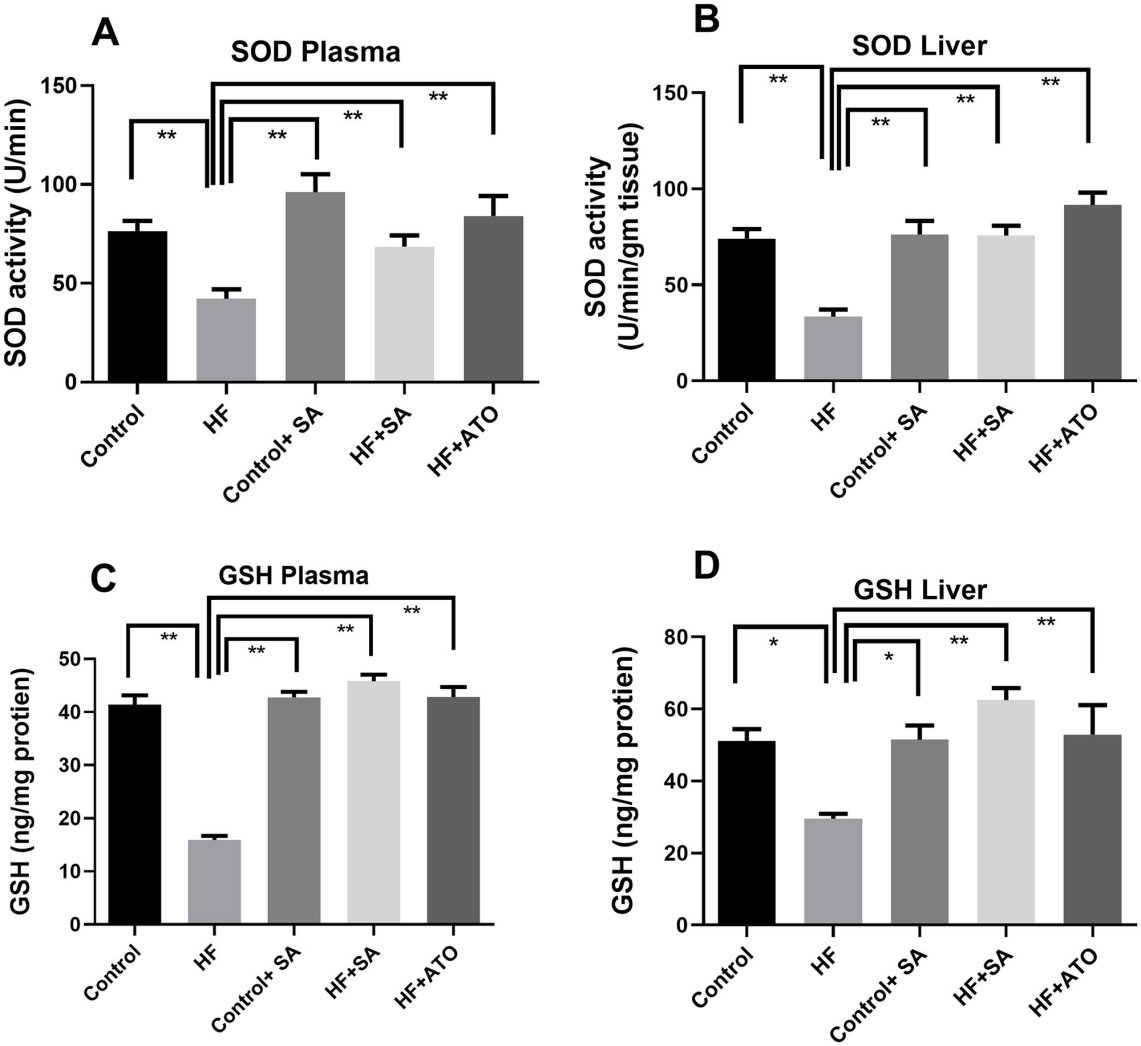
Effect of *Senna alexandrina* leaf supplementation on A. SOD in Plasma; B. SOD in Liver; C. GSH in Plasma and D. GSH in Liver in high fat diet induced obese rats. Values are presented as mean ± SEM, n = 8. One-way ANOVA followed by Tukey’s multiple comparisons test was done for statistical comparison. * represents p≤0.05; ** represents p≤0.001.

SOD activities in both plasma and liver were found to be significantly (p ≤ 0.01) lower in rats in the HF group than those in the control group (Fig 5A and 5B). This reduction in activity was restored by *S*. *alexandrina* treatment as seen in the plasma and liver of rats from the HF + SA group (Fig 5A and 5B), which had significantly greater SOD activity compared to those from the HF group (p ≤ 0.01). Atorvastatin treatment in HF + ATO rats also increased SOD activity in a similar manner. Moreover, *S*. *alexandrina* supplementation did not change SOD activity in liver tissue, but slightly altered its activity in the plasma of control + SA rats (Fig 5B and 5A).

Similarly, GSH concentration in plasma (p ≤ 0.01) and liver tissue (p ≤ 0.05) of HF rats were depleted in comparison with that of control rats (Fig 5C and 5D). This was restored by *S*. *alexandrina* supplementation as GSH activity in plasma and liver tissue of HF + SA rats was significantly higher (p ≤ 0.01) than that of rats from the HF group (Fig 5C and 5D). HF + ATO rats also showed increased GSH concentrations in both plasma and tissue (Fig 5C and 5D) compared to HF rats. However, *S*. *alexandrina* supplementation did not alter GSH concentrations in the rats of the control group (Fig 5C and 5D). Therefore, *S*. *alexandrina* inhibits the depletion of antioxidant enzymes seen HF diet-fed rats.

### Effect of *S*. *alexandrina* leaf powder supplementation on lipid profiles

**Fig 6:**
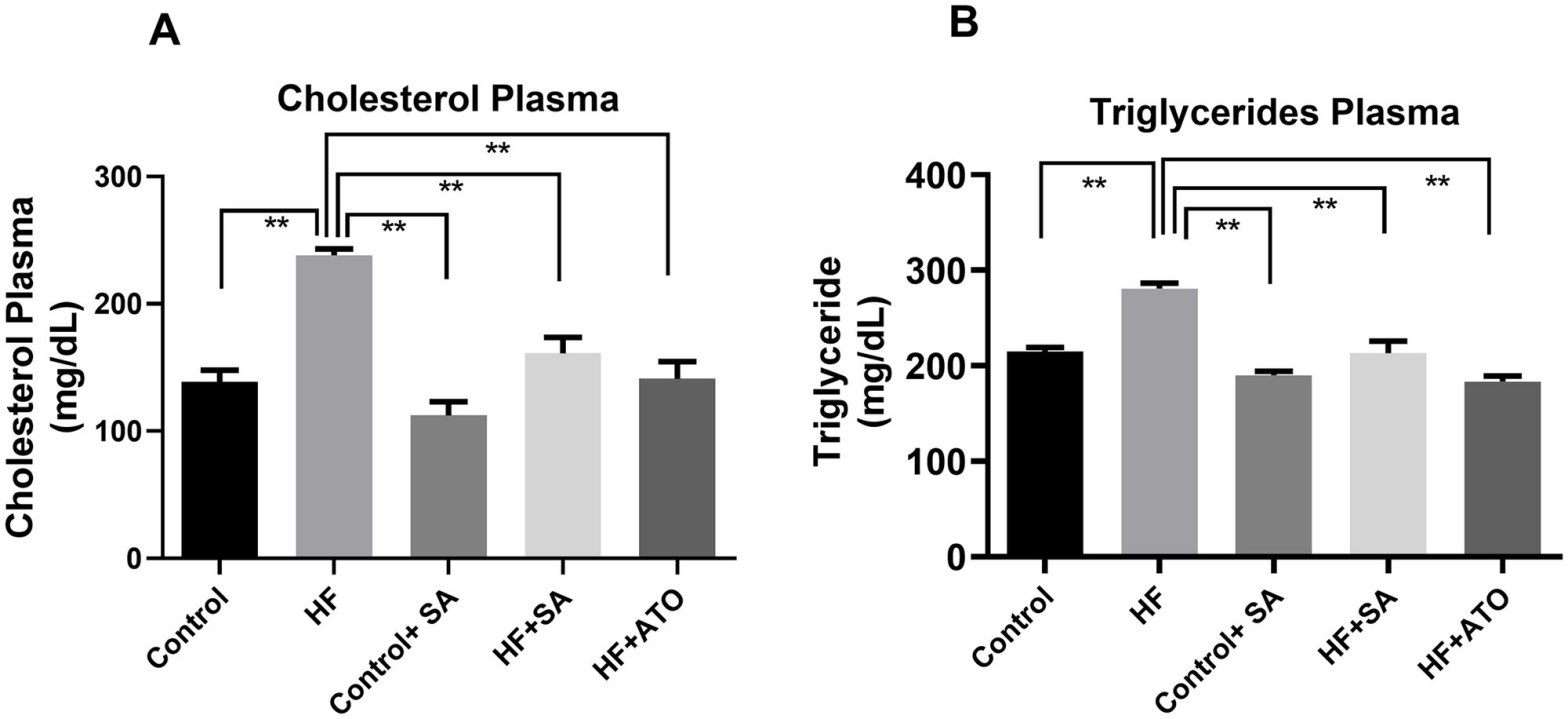
Effect of *Senna alexandrina* leaf powder supplementation on lipid profiles in high fat diet induced obese rats. A. Cholesterol in Plasma. B. Triglycerides in Plasma. Mean ± standard error of mean (SEM) was used to express all values, where n = 8. Statistical analysis was done by one-way ANOVA and comparisons among the groups were done following Tukey’s multiple comparisons test. ** represents p≤0.001.

We measured the total cholesterol and triglyceride levels in plasma to evaluate the lipid-lowering effects of *S*. *alexandrina* leaves on HF rats. We observed that total cholesterol and triglyceride levels in plasma were significantly elevated (p ≤ 0.01) in the HF group compared to the control group (Fig 6A and 6B). *S*. *alexandrina* leaf powder supplementation resulted in significantly lower (p ≤ 0.01) plasma total cholesterol and triglyceride levels in the HF + SA group compared to the HF group (Fig 6A and 6B). Similar trend was observed in cholesterol and triglyceride levels in HF + ATO (Fig 6A and 6B). Moreover, the control + SA group exhibited similar levels of cholesterol and triglyceride as the control group.

### Effect of *S*. *alexandrina* leaf powder supplementation on fat metabolizing gene expression in liver

**Fig 7:**
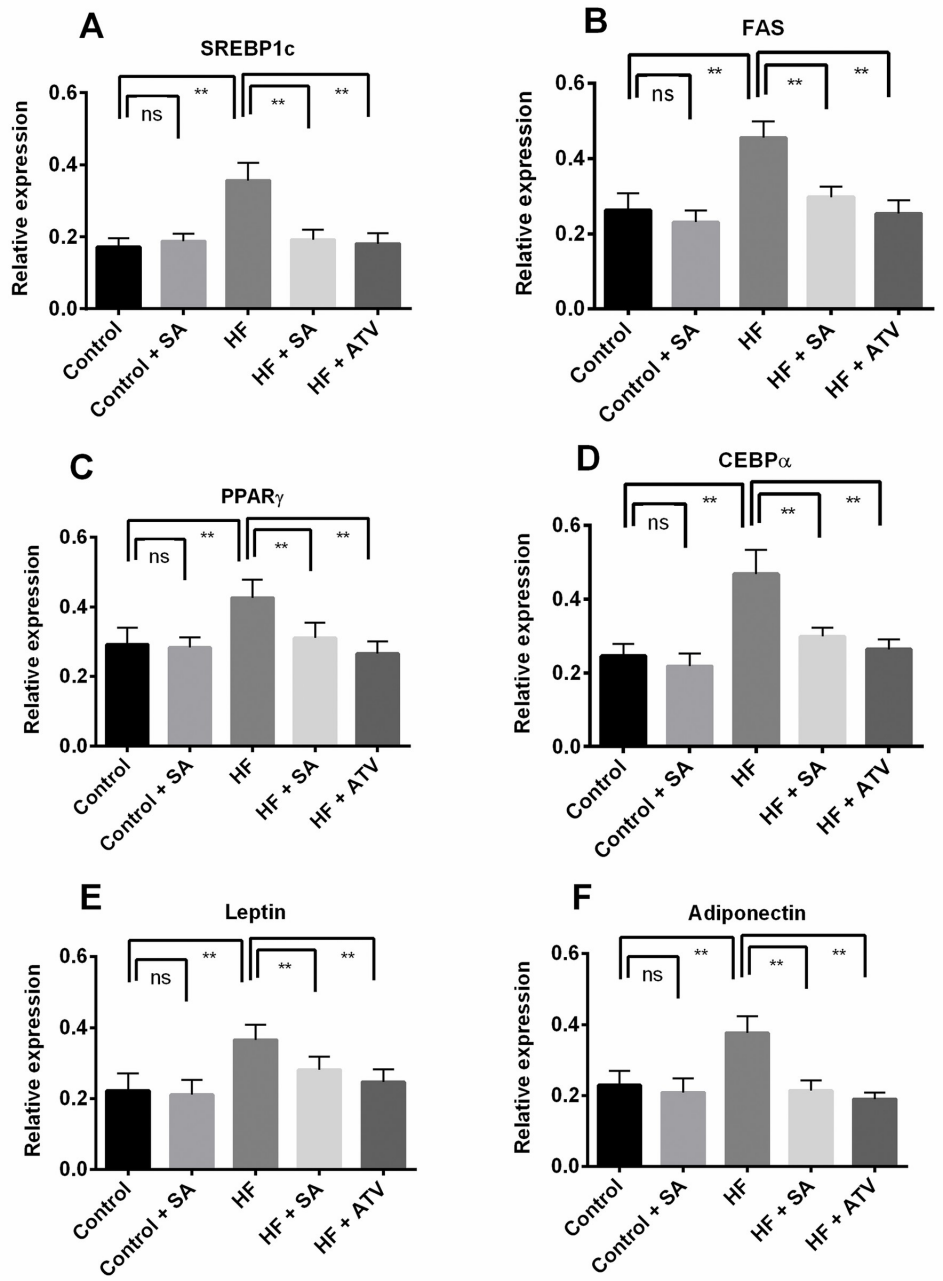
Effect of *Senna alexandrina* leaf supplementation on Fat metabolizing genes expression in liver of high-fat diet induced obese rats. A. Sterol regulatoryi element binding protein 1c (SREBP1c). B. Fatty acid synthase (FAS). C. Peroxisome proliferator activated receptor (PPARγ). D. CCAAT enhancer binding protein alpha (CEBPα). E. Leptin. F. Adiponectin. Values are presented as mean ± SD, n = 6. One-way ANOVA followed by Tukey’s multiple comparisons test was done for statistical comparison. Values are considered significance at p≤0.05. ** represents p≤0.001.

The expression of lipogenesis-related genes in liver tissue was studied to understand the effects of *S*. *alexandrina* leaf powder supplementation on lipid metabolism. Therefore, the relative expression of some fat metabolizing genes in each group is shown in Fig 7.

Compared to the rats in the control group, the HF rats had elevated mRNA levels (p ≤ 0.05) of adipogenic markers such as *SREBP1c*, *FAS*, *PPARγ*, and *CEBPα* (Fig 7A, 7B, 7C and 7D). In contrast, the mRNA expression of these genes in *S*. *alexandrina* treated rats (HF + SA) was lower than that in the HF group (Fig 7A, 7B, 7C and 7D) and comparable to that in HF + ATO group (Fig 7A, 7B, 7C and 7D). Relative gene expression of leptin and adiponectin was also elevated in the HF rats compared to the control rats (Fig 7E and 7F). mRNA expression significantly reduced (p ≤ 0.05) in HF + SA rats compared to the HF rats and was similar to the effects of atorvastatin treatment. However, no significant change in relative expression due to *S*. *alexandrina* leaf powder supplementation was found in the rats of the control + SA group (Fig 7).

### Effect of *S*. *alexandrina* leaf powder supplementation in liver on inflammatory gene expression

**Fig 8:**
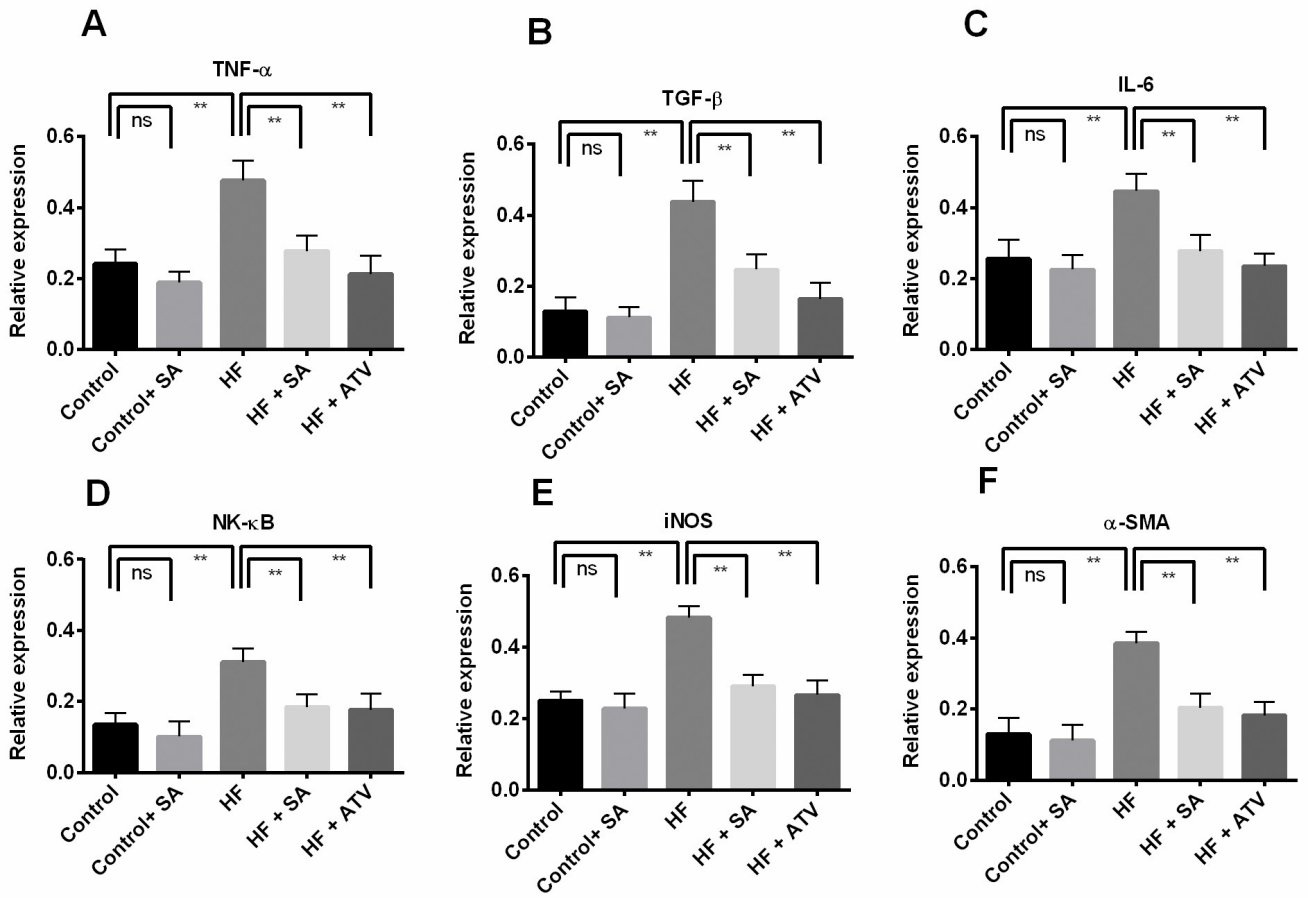
Effect of *Senna alexandrina* leaf powder supplementation on inflammatory genes expression in liver of high fat diet induced obese rats. Here, A. Tumor necrosis factor alpha (TNF-α); B. Transforming growth factor beta (TGF-β); C. Interleukin-6 (IL-6); D. Nuclear factor kappa B (NK-κB); E. Inducible nitric oxide synthase (iNOS); F. Alpha smooth muscle actin (α-SMA). Values are presented as mean ± SD, n = 6. One-way ANOVA followed by Tukey’s multiple comparisons test was done for statistical comparison. ** represents p≤0.001.

To evaluate whether *S*. *alexandrina* leaf powder supplementation altered hepatic inflammation, we studied the relative expression of some genes related to inflammation or hepatic injury. The expression of these genes is displayed in Fig 8.

The results indicated that the mRNA levels of pro-inflammatory and cytokine genes, such as *TNF-α*, *TGF-β*, and *IL-6*, were significantly (p ≤ 0.05) higher in the HF group than in the control group (Fig 8A, 8B and 8C). However, the mRNA levels of these genes lowered (p ≤ 0.05) in the HF + SA group compared to the HF group. Atorvastatin treatment showed similar effects in HF + ATO rats compared to the HF group (Fig 8A, 8B and 8C). The relative expression of *NF-κB* and *iNOS* was also higher (p ≤ 0.05) in the rats of the HF group compared to those of the control group (Fig 8D and 8E). In the HF + SA group, mRNA levels of *NF-κB* and *iNOS* were lower (p ≤ 0.05) than those in the HF group, and similar trends were seen in the HF + ATO group (Fig 8D and 8E). Furthermore, to evaluate the effects of *S*. *alexandrina* on high-fat diet-induced hepatic fibrosis, we also analyzed the expression of *α-SMA* (a marker of myofibroblastic hepatic stellate cells). The expression of this gene was significantly (p ≤ 0.05) higher in the HF group than in the control group (Fig 8F). However, *S*. *alexandrina* supplementation as well as atorvastatin treatment lowered the expression of this gene in HF + SA and HF + ATO groups, respectively, compared to that in HF group (Fig 8F). Nevertheless, *S*. *alexandrina* supplementation did not alter the mRNA levels of these genes in control rats (Fig 8).

### Effects of *S*. *alexandrina* leaf powder supplementation on antioxidant enzyme gene expression in liver

**Fig 9:**
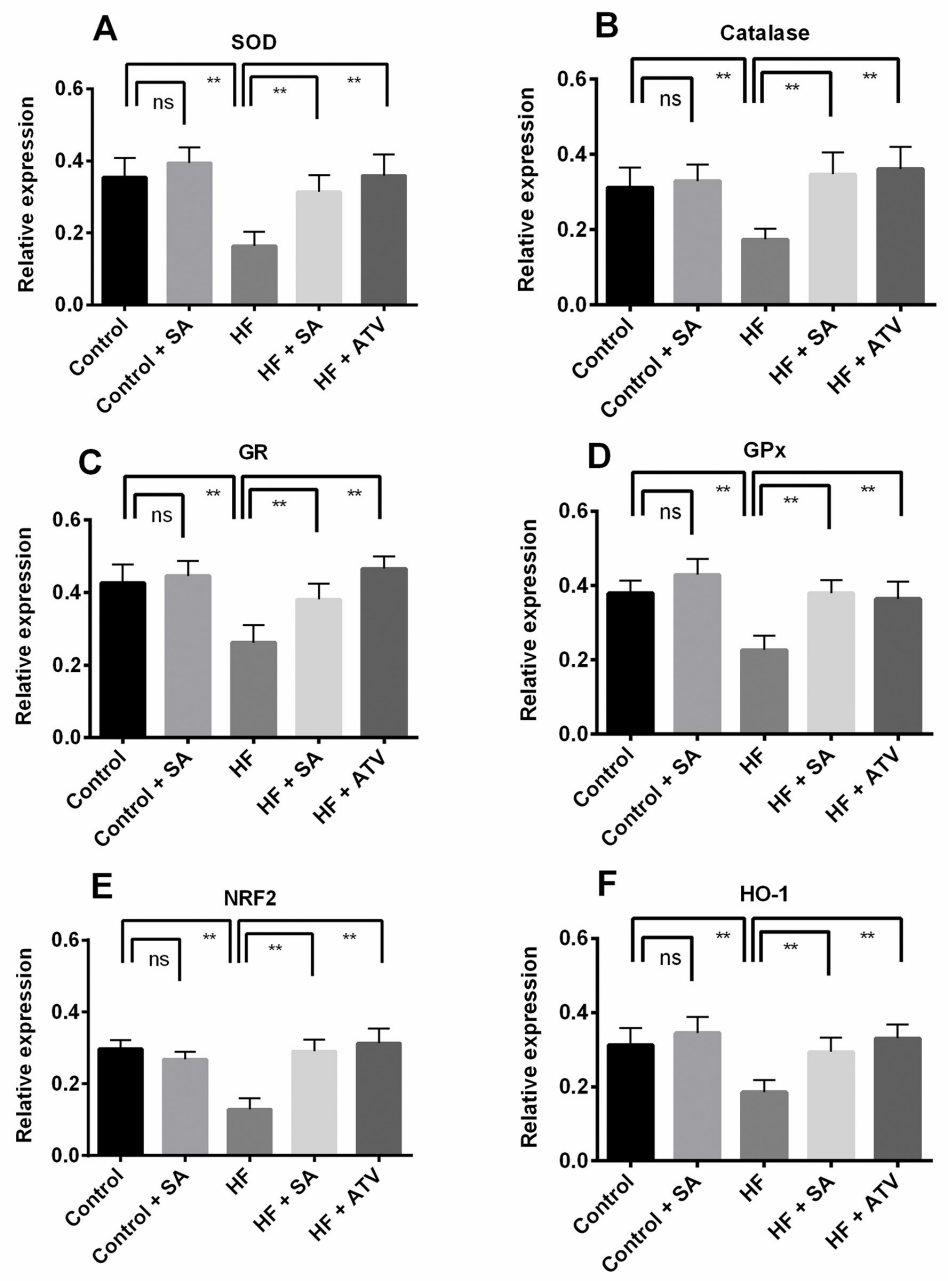
Effect of *Senna alexandrina* leaf powder supplementation on antioxidant enzyme activity genes expression in liver of high fat diet induced obese rats. Here, A. Super oxide dismutase (SOD); B. Catalase; C. Glutathione reductase (GR); D. Glutathione peroxidase (GP_x_); E. Nuclear factor erythroid 2-related factor 2 (NRF 2); F. Heme oxygenase-1 (HO-1). Values are presented as mean ± SD, n = 6. One-way ANOVA followed by Tukey’s multiple comparisons test was done for statistical comparison. ** represents p≤0.001.

Previous studies as well as the results of antioxidant enzyme activities in this study in both plasma and liver tissue suggest that these enzyme activities are lower in obese individuals. For better understanding, we also studied the expression of some antioxidant genes, such as *SOD*, *CAT*, *GR*, and *GPx*. The expression of these genes is shown in Fig 9.

Our results indicated that the relative expression of the genes *SOD* and *CAT* was significantly (p ≤ 0.05) lower in the HF group compared to that the control group (Fig 9A and 9B). However, rats in the HF + SA group showed higher relative expression of these genes (p ≤ 0.05) compared to those in the HF group. In comparison with the HF group, the HF + ATO group also showed greater expression of these genes, similar to HF + SA group (Fig 9A and 9B). We observed a similar trend for the other two antioxidant enzymes *GR* and *GPx* as well. The mRNA transcript levels of these genes were found to be significantly (p ≤ 0.05) lower in HF rats than in control rats (Fig 9C and 9D), but *S*. *alexandrina* leaf powder supplementation resulted in elevated (p ≤ 0.05) mRNA transcript levels, similar to that seen in control group.

To determine whether the NRF2/HO-1 signaling pathway is involved in the protective effects of *S*. *alexandrina* against oxidative stress, we also studied the mRNA expression of *NRF2* and *HO-1*. The results indicated that rats of the HF group had significantly (p ≤ 0.05) lower *NRF2* and *HO-1* mRNA expression compared with the rats of the control group (Fig 9E and 9F). However, the expression of these genes in the HF + SA group was significantly higher (p ≤ 0.05) than that in the HF group. Atorvastatin treatment with HF diet also upregulated the expression of these genes compared to the HF group, and this upregulation was very similar to that observed in the HF + SA group (Fig 9E and 9F). Importantly, *S*. *alexandrina* supplementation did not change the mRNA levels of antioxidant genes in control rats (Fig 9).

### Impact of *S*. *alexandrina* leaf powder supplementation on histology of liver and intestine

**Fig 10:**
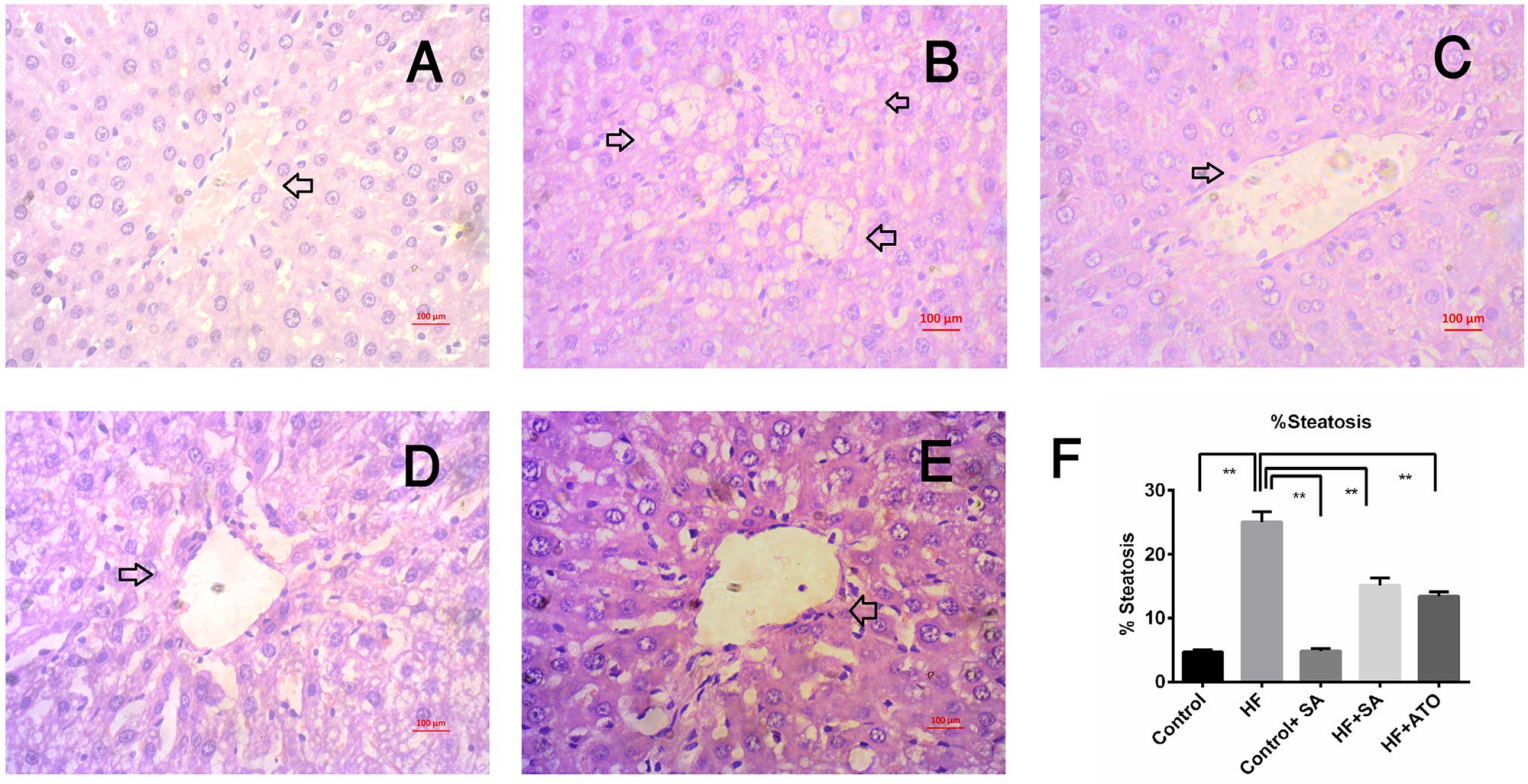
Effect of *Senna alexandrina* leaf powder supplementation on lipid accumulation in liver of high-fat diet-fed rats. A for control; B for HF; C for Control+SA; D for HF+SA; E for HF+ATO group and F for % Steatosis. Magnifications 40X.

Fig 10A to 10E depicts the histology of the liver through hematoxylin and eosin staining of organs of the five groups. Rats of the control group (Fig 10A) showed no steatosis and fat droplets compared to HF rats (Fig 10B). However, HF + SA rats showed less fat droplets deposition and steatosis compared to HF rats (Fig 10D). HF + ATO also showed lower steatosis score compared to HF rats (Fig 10E). Control + SA rats showed normal architecture (Fig 10C) as in control rats.

**Fig 11:**
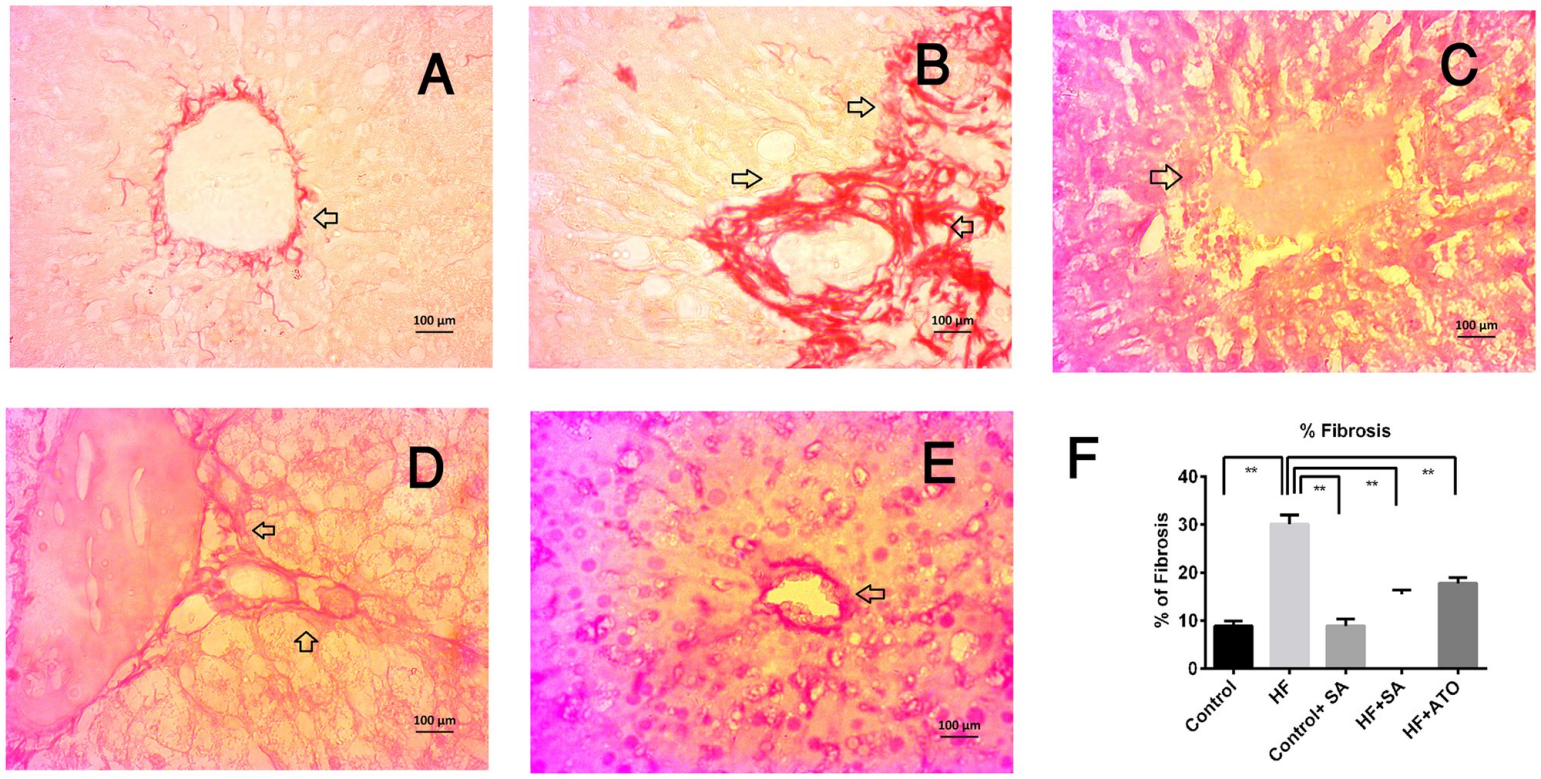
Effect of *Senna alexandrina* leaf powder supplementation on fibrosis in liver of high-fat diet-fed rats. A for control; B for HF; C for Control+SA; D for HF+SA; E for HF+ATO group and F for % Fibrosis. Magnifications 40X.

Fig 11A to 11E depicts Sirius red staining of the liver of rats from each group. HF rats (Fig 11B) showed increased collagen deposition compared to control rats (Fig 11A), but those treated with *S*. *alexandrina* leaf powder supplementation at the same time showed (Fig 11D) much less collagen deposition compared to HF rats (Fig 11B). Moreover, collagen deposition in HF + ATO group (Fig 11E) was also decresed compared to HF rats. *S*. *alexandrina* leaf powder supplementation treatment in control + SA rats resulted in normal collagen deposition (Fig 11C).

**Fig 12:**
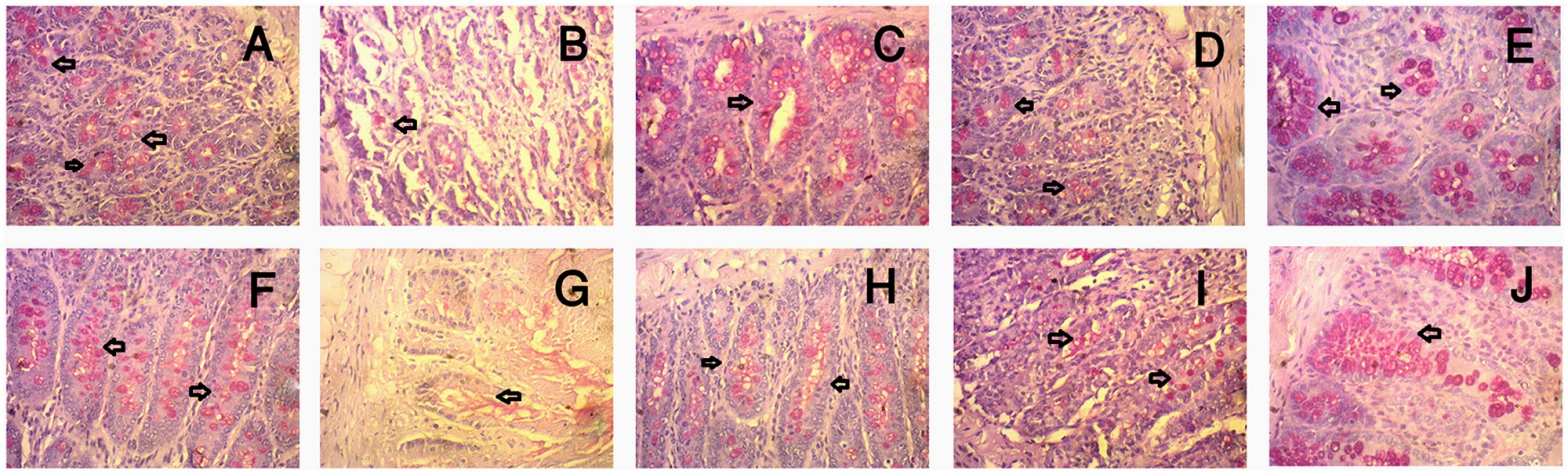
Effect of *Senna alexandrina* leaves powder supplementation on goblet cells population in crypt (upper panel) and villi (lower panel) region of intestine in high-fat diet-fed rats. A and F for control; B and G for HF; C and H for Control+SA; D and I for HF+SA and E and J for HF+ATO group. Magnifications 40X.

Fig 12 shows the histology of the intestine of rats across groups. Fig 12A to 12E depicts crypts while Fig 12F to 12J indicates villi. Control rats (Fig 12A and 12F) showed the presence of more goblet cells in crypt and villi compared to HF rats (Fig 12B and 12G). Moreover, HF rats showed (Fig 12B and 12G) less goblet cells in crypt and villi compared to HF + SA (Fig 12D and 12I). HF + ATO rats (Fig 12E and 12J) showed similar numbers of goblet cells in crypt and villi as that seen in the rats of HF + SA group (Fig 12D and 12I). Additionally, control + SA rats did not show any changes in the number of goblet cells in the crypt and villi when subjected to treatment with *S*. *alexandrina* leaf powder (Fig 12C and 12H).

## Discussion

Obesity is a major risk factor for several illnesses and mortality. The statistics of obesity in the world over the last two decades are a cause for concern [29]. The rate of white adipose tissue accumulation is elevated in obesity. Obesity leads to a cluster of diseases such as glucose intolerance, hyperlipidemia, NAFLD, hypertension, and cardiovascular dysfunction [30, 31]. HF diet-induced obesity models in animals are commonly studied because these animal models show similar complications as seen in humans. In this study, we developed an obese rat model using a HF diet that resulted in the development of dyslipidemia and NAFLD. This investigation also revealed the impact of senna leaf powder supplementation on oxidative stress, inflammation, and related complications in the liver of HF diet-fed rats.

Provision of HF diet results in increased plasma lipid and glucose levels as well as increased triglyceride and cholesterol levels in rats. This increase in glucose and lipid profiles is caused by saturated fats, which are present in HF diet [32]. Increased serum glucose concentrations were observed in HF rats, as shown in a previous study [33]. After glucose challenge, HF rats were unable to utilize glucose properly to establish homeostasis and develop glucose intolerance. Aminoglycosides (especially sennoside A and B) in senna have been reported to have antihyperglycemic activity [13, 34]. The aqueous extract of senna improves metabolic abnormalities linked with diabetes and reduces chronic hyperglycemia-related complications in streptozotocin-induced diabetic rats [17, 35]. In our experiment, glucose utilization was improved by *S*. *alexandrina* leaf powder supplementation, which is supported by a previous study [36].

Hepatic condition can be assessed by measuring the activities of hepatic transaminase enzymes such as ALT, AST, and ALP in the blood stream. The increased activities of these enzymes in the blood are indicative of liver damage or the initiation of hepatic deterioration. Early oxidative stress in tissues plays an important role in this process. In the present study, we found elevated activities of these liver marker enzymes in the HF group compared to the control group. This finding is also supported by previous scientific studies [37, 38]. However, supplementation with *S*. *alexandrina* leaves reduced the activities of liver function marker enzymes. Oxidative stress indicates increased levels of reactive oxygen species (ROS) in cells. Different mechanisms are involved in generating ROS in obesity including α and β oxidation of fatty acids by peroxisomes and excess consumption of oxygen by the respiratory chain in mitochondria, which propagates free radicals in a cluster of reactions called oxidative phosphorylation [39–41].

Previous report also showed that mitochondrial dysfunction may develop oxidative stress in liver of HF diet fed rats [5]. This study as well as previous studies, reported that the levels of oxidative stress markers were elevated in plasma and tissue in animals fed with HF diet [5]. However, *S*. *alexandrina* leaf powder supplementation reduced the levels of all oxidative stress markers in HF diet fed rats. A previous report suggested that the ethanol extract of senna has high antioxidant properties [42]. A wide variety of flavonoids and phenolic acids, such as gallic acid, caffeic acid, and vanillic acid, are found at high levels in different parts of senna [43, 44]. The human body usually neutralizes ROS through enzymatic (SOD, GPx, and CAT) and non-enzymatic (reduced GSH) means. However, when these ROS preventing agents are unable to neutralize excess ROS in obese subjects, cellular as well as molecular changes are seen. In the present study, we found significantly lower activity of SOD and GSH in plasma and liver tissue in HF group compared to that in control group. The activity of these antioxidant enzymes was higher in *S*. *alexandrina* leaf powder supplemented HF diet fed rats than that of the HF group, which demonstrates that *S*. *alexandrina* leaf powder improves the cell reinforcement limit by expanding ROS prevention.

Furthermore, we studied the expression of some antioxidant genes, such as *SOD*, *CAT*, *GR*, and *GPx*. The results indicated that the relative expression of these genes was significantly lower in the HF group than in the control group. *S*. *alexandrina* leaf powder supplementation in HF diet fed rats up-regulates the relative expression of these genes. Moreover, the results suggest that *S*. *alexandrina* leaf powder supplementation may improve hepatic oxidative stress by activating the *Nrf2* gene, which is a master regulator of oxidative stress related gene expression. This findings are in consonance with the previous reports suggested that *Nrf2* gene expression may decline in liver due to HF diet which may be augmented by docosahexaenoic acid and hydroxytyrosol administration and improved the mitochondrial function [5, 45]. The relative expression of *Nrf2* and its target genes (such as *HO-1*) is up-regulated in the HF diet fed rats supplemented with *S*. *alexandrina* leaf powder compared to the HF diet fed rats alone, which is consistent with a previous study [46].

The present study also provides evidence that *S*. *alexandrina* leaf powder supplementation reduces the levels of triglyceride and cholesterol in HF diet-fed rats. Previous studies revealed that the expression of lipogenic genes (such as *FAS*, *SREBP1c*, *IR*) and lipid metabolism genes (like *PPARγ*), are affected in the hepatic tissue of obese subjects [47, 48]. In the present study, we found that HF rats had elevated mRNA levels of adipocyte markers such as *SREBP1c*, *FAS*, *PPARγ*, and *CEBPα* compared to control rats. However, *S*. *alexandrina* leaf powder supplementation markedly downregulated the mRNA expression of these genes. Many hormones, such as leptin and adiponectin, play vital roles in fat metabolism in the human body. Hence, senna leaf powder supplementation attenuated gene expression related to fat metabolism.

Moreover, to evaluate whether *S*. *alexandrina* leaf powder supplementation altered hepatic inflammation, we also studied the relative expression of some genes related to inflammation or hepatic injury. The results indicated that the mRNA levels of pro-inflammatory and cytokine genes, such as *TNF-α*, *TGF-β*, and *IL-6*, which are modulated by NF-kB, a redox-sensitive transcription factor, were significantly higher in the HF group than in the control group. It has been suggested that the saturated fatty acid present in the HF diet is responsible for the inflammatory response through NF-кB activation in liver [49]. This result is also consistent with previous scientific studies [49, 50]. However, *S*. *alexandrina* leaf powder supplementation lowered the mRNA levels of these pro-inflammatory and inflammatory genes.

The final events of hepatic cell damage triggered by oxidative stress, such as fibrosis and inflammatory cell infiltration, are observed in HF diet fed rats. Several food supplements are rich in antioxidants that may ameliorate fibrosis and inflammatory cell infiltration. Hepatic stellate cells are the main source of collagen deposition in the extracellular matrix and are activated by oxidative stress. Therefore, the avoidance of oxidative stress might be a good preventive measure for fibrosis. In this study, *S*. *alexandrina* leaf powder supplementation ameliorated inflammation and diminished the deposition of scleroprotein collagen within the liver of HF diet fed rats.

This investigation revealed the beneficial effects of *S*. *alexandrina* leaf powder supplementation in HF diet-fed rats. However, long-term use of senna may have some deleterious effects that are mostly related to its laxative effect. Senna powder supplementation in control rats in this investigation showed no adverse reaction in terms of the activity of hepatic trans-aminases, which were not altered compared to control rats. Moreover, senna powder supplementation in control rats resulted in decreased body weight, which was due to the decreased fat deposition in control rats. Previous studies have also investigated the toxicity of senna powder. One study showed that treatment with *S*. *alexandrina* for five weeks did not produce significant carcinogenicity in C57BL/6N mice [51]. Another study on mice showed that forty weeks of treatment with *S*. *alexandrina* did not result in any neoplastic changes [51]. Moreover, there is no convincing evidence of altered structural and functional activities of the smooth intestinal muscle after long-term use of *S*. *alexandrina* [52]. This study also revealed that the microstructural environment was unchanged in the intestine of rats after senna powder supplementation, whereas a de-organized structure of intestine and intestinal brush border was observed in HF rats. Fibrosis and fewer goblet cells were observed in the crypt region of HF rats, but the crypt structure and intestinal microvilli were restored by *S*. *alexandrina* leaf powder supplementation. A previous report concluded that *S*. *alexandrina* at 300 mg/Kg per day for two continuous years may not result in carcinogenicity in rats and does not pose any risk of genotoxicity [52, 53]. A previous acute toxicological investigation of *Senna alata* extract also revealed no mortality at the maximum dose of 10 g/kg after 24 h in Wistar rats [54].

In conclusion, the present study revealed that the supplementation of senna leaf powder impedes obesity and ameliorates glucose intolerance, oxidative stress, and fat metabolism in HF diet-induced obese rats. This study was primarily aimed at establishing senna leaf powder supplementation for treating obesity and other related complications. However, toxicological studies and clinical trials must be conducted before this leaf powder may be used as an alternative treatment approach against obesity and its related metabolic syndrome.
